# Topical steroid withdrawal: dissecting the controversy

**DOI:** 10.3389/fmed.2026.1786331

**Published:** 2026-03-17

**Authors:** Ian A. Myles, Grace Ratley

**Affiliations:** Laboratory of Clinical Immunology and Microbiology (LCIM), Epithelial Therapeutics Unit, National Institute of Allergy and Infectious Diseases (NIAID), National Institutes of Health (NIH), Bethesda, MD, United States

**Keywords:** atopic dermatitis, corticosteroids, Eczema, steroid, topical steroid withdrawal (TSW)

## Abstract

In the past few years, topical steroid withdrawal (TSW) has received increasing recognition from research and regulatory agencies. Delphi protocols have solidified the clinical features and treatment options recognized by clinicians. Pilot studies elucidating the mechanisms of TSW pathology have been performed. Meanwhile, regulatory bodies have updated the topical corticosteroids (TCS) warning labels to include TSW. And yet, the idea that TSW is distinct from the underlying dermopathies which conferred the TCS exposure remains debated by some. This narrative review aims to help providers better understand the origins of, and current state of, the controversy surrounding TSW. We review the history of TSW claims and counter claims, focusing on sources found in the medical literature. Having presented the development of TSW skepticism, we next detail the errors in the three main examples of TSW denial: ignoring the syndromic nature of TSW to focus on skin pathology which overlaps with other dermopathies; systematically devaluing patient-reported symptoms; and circular logic asserting that studies into TSW cannot be conducted without first establishing diagnostic criteria requiring studies to obtain. Finally, we present an updated assessment in the knowledge gaps surrounding TSW. We hope that this report will help providers recognize TSW, especially in contrasting the disorder with the dermopathies which have similar presentations of cutaneous symptoms. Overall, we believe this report will help providers better reassure patients to whom the diagnosis of TSW should not be applied, better council patient on the risks of TCS, and better provide trauma-informed care for patients who with TSW.

## Introduction

In the past few years, topical steroid withdrawal (TSW) has received increasing recognition from research and regulatory agencies. Delphi protocols have solidified the clinical features which were presented in the historical case reports and case series (1). Pilot studies elucidating the mechanisms of TSW pathology have been performed (2). While regulatory bodies have updated official labeling for topical corticosteroids (TCS) (3). And yet, consideration of the diagnosis as being distinct from other dermatologic diseases is still met with doubts from some academicians and healthcare providers. While these doubts are seen as healthy skepticism by academia, the same doubts are viewed as invalidating by the patients who feel wronged by the health care system.

With this narrative review, we aimed to identify and summarize the criticisms against seeing TSW as a distinct entity. We began by outlining the historical process by which the disagreement between academics and advocates developed. Next, we describe the main features of TSW skepticism and assess the validity of these claims against the historic record and most recent discoveries. Having dissected the denialism, we present a more accurate identification of the TSW knowledge gaps which require dedicated research. Finally, we outline general guidelines that practitioners should follow to bridge the gap between providers and patients. We hope that understanding how and why TSW was, and still is, overlooked may offer means of improving partnerships in patient care.

## Historical perspective

The first academic use of the term “topical steroid withdrawal” came in two related publications detailing pathologic reactions occurring after discontinuation of topical corticosteroids (TCS) in 1976 (4) and 1977 (5). Although each report focused on intracranial hypertension, the skin was described as “scaly and desquamating with a slightly offensive smell” (4). The mechanism proposed for each was the well-known suppression of the hypothalamic-pituitary-adrenal (HPA) axis occurring after systemic glucocorticoid exposure. This creates a form of physiologic dependence on exogenous glucocorticoids to maintain homeostasis; when glucocorticoids are removed the patient is unable to maintain adequate HPA signaling. The first reports of what are now known to be direct TCS toxicities (such as telangiectasias, vasodilation, and skin thinning) also appeared (4, 6, 7).

### Initial descriptions

In 1979, and again in 1993, two reports used the term “topical corticosteroid addiction” to describe patients with overuse of high-potency steroids on their face, leading to perioral dermatitis or rebound telangiectasias (8, 9). The reports used the term “addiction” in response to the patients' obsessive focus on facial aesthetics of clear skin (8) or as a way of describing rebound phenomena. Thus, these reports did not invoke mechanisms of true physiologic addiction. Then in 1999, and again in 2003, Dr. Marvin Rapaport described 100 cases of patients who were using TCS for eyelid dermopathies who experienced symptoms on the entire face after discontinuation (10, 11); he used the terms “red face syndrome” and “red burning skin syndrome.” Later, Dr. Rapaport co-founded the International Topical Steroid Awareness Network (ITSAN) to bring awareness to under recognized complications of TCS.

In 2014, Fukaya et al. (12) specifically defined “topical steroid addiction” (TSA) as when the “skin develops more severe or diverse skin manifestations after the withdrawal from TCS than at preapplication”. This expanded Dr. Rapaport's definition beyond facial disease. In both case series the TSW/TSA-related erythema extended beyond the borders of the previous eczematous lesions: for Dr. Rapaport, that involved a shift from the eyelids to the face whereas Dr. Fukaya described changes from traditional AD distribution to the entire body. Other specific findings which distinguished TSW/TSA from AD included: itch that can no longer be controlled with scratching (later termed unsatisfiable itch); profound desquamation following the acute redness phase; reactivity to sweating; temperature dysregulation; and pronounced depression (12). Fukaya's report remains the only one which attempted to assess the incidence of TSW among chronic TCS users, extrapolating from surveys on uncontrolled AD to derive an estimate of 12% (12). The report was also the first to take issue with the American Academy of Dermatology (AAD) guidelines advocating for a “proactive approach,” which argued for daily TCS use for patients who experience AD recurrence upon discontinuation (13). Dr. Fukaya's group instead suggested using non-steroidal controller medications for patients who used TCS more than 14 days per month for symptom control (12, 14).

### The second wave

In 2015, the National Eczema Association sponsored a study investigating TSW. While well intended, the report unfortunately opted to only assess TSW from the academic publications available at that time. Collecting case reports of adverse reactions to TCS led the consortium to conclude that most cases involved patients with disease and TCS exposure limited to their face (15). Patients who used moderate to high potency TCS on their face or genitals, against medical advice and package inserts, were reported to have symptoms of irritation directly attributable to TCS exposure. The likely mechanism was mediated by skin thinning leading to eroded barrier function. Similar irritation was seen in patients with contact dermatitis from allergic reactions to the TCS or their excipients. However, in addition to excluding many of the prior case reports, the project neglected to solicit input from patient advocates to characterize symptoms that may differentiate TSW from these well-established TCS complications. The report also overlooked the fact that failing to identify published cases such as those outlined by Fukaya et al. (12) might have been a reflection of a deficiency in the literature rather than a lack of such cases.

Unfortunately, the field was left with a publication with many high-profile authors which suggested that TSW was more attributable to patients failing to follow instructions than providers failing to recognize a previously underreported syndrome. As an example of misinterpretation, subsequent AAD guidelines pointed to the NEA review as having “… analyzed published case series and reports and deemed the strength of the evidence regarding TSA/TSW to be low to very low” (16). While technically correct, this framing overlooks that the GRADE system used to assess the evidence designates all observational data as low quality (17) and omits that the conclusion of the report was that TSW “…is distinct from other well-described TCS adverse effects” (15). Therefore, noting that the “low quality” of evidence was meant to advocate that “well-designed, prospective studies are needed” (15); it was not meant to suggest that the “absence of evidence” indicated the “evidence of absence” (18) for TSW.

In the 8 years following the NEA report, eighteen additional case reports and case series were published, including many by Dr. Belinda Sheary, Dr. Eric Simpson, and Dr. Peter Lio (19–37); each case presented a similar picture of expansile dermatitis and neurogenic symptoms spreading beyond the original dermopathy. In 2023, the first large-scale patient survey was conducted by ITSAN and the Allergy & Asthma Network. The survey quantified the symptoms reported by patients with self-diagnosed TSW and identified edematous folds on flexor surfaces (termed “elephant skin”) as another TSW-distinguishing symptom (38). A shared feature of many of these case series was the clearance of skin symptoms (or return to prior skin symptoms) with the prolonged avoidance of TCS. While the natural history of AD might include resolution of skin symptoms over time for some (39), improvement of severe AD without anti-inflammatory treatments would be unexpected (especially in the presence of the additional, distinguishing symptoms).

Importantly, despite the mounting case reports and patient data, only two mechanistic evaluations of TSW were performed prior to 2024 (40, 41). These publications proposed that cutaneous downregulation of the glucocorticoid receptor was responsible for TSW, however the lack of isotype controls render the immunohistochemistry difficult to interpret. The technical flaws in the mechanistic work further highlighted that a disorder prevalent enough to beget an advocacy group with tens of thousands of members had not received detailed assessments compared to other skin diseases.

### Landing upon deaf ears

Despite the complete lack of mechanistic assessment, the academic response to both the mounting case reports and patient survey was to ask, “Should we worry?” (42). The criticisms leveled were a distillation of those who doubted TSW was a distinct dermopathy: the patients presented/surveyed were self-selected; TSW lacked diagnostic criteria; patients are often exposed to more than one medication class; and allergic and/or toxic reactions may also present with dermatitis.

The questions of whether worry was justified followed upon the 2008 study of eight patients with AD who were asked to apply TCS twice daily for 4 weeks surreptitiously monitored TCS use via microprocessors in the container cap (43). Six of the 7 participants who completed the study demonstrated at least 60% adherence – meaning they averaged greater than daily application of the TCS for a month. Finding that greater TCS use associated with reduced disease severity led the authors to conclude that patients with uncontrolled AD should be suspected of poor adherence. While there is no gold-standard definition of “non-adherence,” many studies define it as complete avoidance of the medication in question (such as failure to fill a prescription) (44), rather than using a twice-daily prescription only once a day. Furthermore, the authors did not solicit or document any complications which might have confounded adherence, especially if use typically lasts much longer than 4 weeks. The overall take away from this, and similar work, was that patients who do not respond to TCS are non-adherent and the non-adherence was due to irrational fear of complications (deemed “steroid phobia”).

Denouncing TSW's existence followed shortly after, in 2014, with academic spread of the concept of “steroid phobia” (45). Some early use of the term “steroid phobia” was intended to relay concerns that patients who might benefit from TCS, but who avoid treatment out of fear of complications, might needlessly suffer. However, the development of the TOPICOP score which aimed to quantify “corticophobia” (46) marked a shift toward presenting patient concerns about TCS as a pathology requiring greater treatment and research rather than the published symptoms generating the hesitance.

Although some of the subsequent reports regarding “corticophobia” advocated that optimal care required acknowledging patient concerns (47), many focus on presenting alternative hypotheses to TSW as a means of warning against TCS avoidance (48–50). As noted at the time (51–54), the fact that TSW shared some features of other dermopathies was never in question. Noting that itch and erythema may also stem from contact dermatitis or that loss of TCS efficacy overtime may be “tachyphylaxis” ignores the descriptions of distinguishing symptoms unique to TSW (such as neurologic abnormalities and “elephant skin”); nor does it explain the rapid change in the severity, nature, or distribution of the patients' previous dermopathy. Furthermore, genuine investigation into the risk factors, mechanisms, and treatments of TSW would likely reduce TCS hesitancy by fostering trust through transparent communication. Instead, the patient community was trying to discuss a syndrome overlaying a chronic dermatitis that needed investigation while the academic literature discussed only the underlying, chronic dermatitis.

### Social media distractions

With few prominent academics providing guidance and no mechanistic research into the nuances of the pathology being performed, the TSW patients more often turned to online communities to share advice (55). This created silos between academia and advocates, allowing for reciprocal radicalization. Academic researchers increasingly accused the patient community of spreading online “misinformation,” one referring to TSW as “a particularly prevalent myth currently being propagated on social media” (56). Between 2022–2025, 12 evaluations of TSW-related social medial posts were published [five using patient comments to inform TSW recognition (55, 57–60) compared to six critical of patient commentary (56, 61–65) and one neutrally noting the increase in online TSW mentions (66)].

One disparaging example chastised patient-generated TikTok content for lacking clear definitions of TSW and noting that “neither cause nor mechanism of the disease were described in the videos” (62). Another assessed social media posts across multiple platforms and concluded that “dermatologists with social media platforms can help combat common misconceptions regarding TCS by posting evidence-based videos” (61). These examples seem misguided given that the scientific literature lacks evidence for clear definitions, mechanistic descriptions, risk factor identification, or incidence rates which could inform “evidenced-based videos” from either patients or providers. Unless claims into TSW are scientifically investigated, there can be no rubric for defining what constitutes “misinformation.”

Videos invoking mechanisms which have low pretest probability (such as claiming TSW is primarily caused by “leaky gut”) may be problematic especially when coupled with financial incentives to sell the viewer nutraceuticals. However, the best method for refuting these claims would be to cite evidenced-based mechanism data; data which academia has not yet produced. For example, three patient-generated YouTube documentaries have been produced about TSW. The first two (67, 68) focused on patient narratives. Even if one wanted to criticize these films as focusing on an uncommon complication of an otherwise safe medication, that does not invalidate the patients' histories. The third documentary, *Still Preventable*, provided commentary on cause, mechanism, and defining characteristics through referencing publications from academic and regulatory bodies (69). The third documentary was the only one to discuss treatment options but contextualized anecdotes with academic citations. Meanwhile, academia produced surveys of whether providers “believed” TSW to be real (70, 71) as if survey results inform factual claims.

Overall, this response from the academic literature fed the narrative among patient advocates that researchers were disinterested in the suffering of patients with TSW and/or prioritized the interests of pharmaceutical companies (55). Patients, in need of targeted treatments and distrustful of pharmaceuticals, turned to alternative therapies (38). This expanded the gap between advocates and academics and reinforced the narrative that only providers were evidenced-based. Meanwhile patients felt blamed for their condition, and TSW was deemed a social media construct (64, 65, 72). Overall, a lack of engagement from more traditional information outlets likely left the patient community with few other outlets other than social media.

### Recent developments

**Figure 1:**
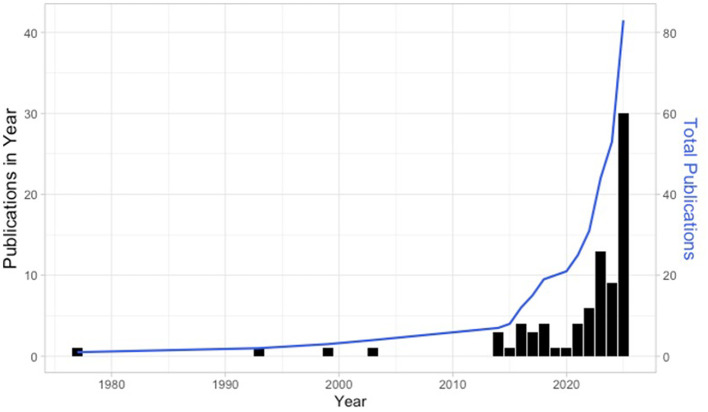
Publications related to Topical Steroid Withdrawal Syndrome. PUBMED was searched for the prompt (“topical steroid withdrawal” OR “topical corticosteroid withdrawal” OR “topical steroid addiction” OR “topical corticosteroid addiction” OR “red burning skin syndrome” OR “steroid addiction”) in January of 2026. The 87 results were screened for relevance at the abstract level. The 85 with specific relevance to TSW are plotted by year. The right Y axis and bars indicate the number of publications within the specified year; the left Y axis and line indicate the running total of publications over the time indicated. Graph was created using R Studio 2024.09.0+375 using ggplot.

2025 contained several advancements in diagnostic, mechanistic, and regulatory understanding of TSW. Over a third of all relevant publications on TSW were published in 2025 (Figure 1). A publication from our group compared previous survey answers between patients with TSW and those with AD but without concerns for TSW (2). Using a penalized regression model, we identified that TSW could be best distinguished from AD by: expansion of disease beyond established body site distribution; new onset burning; flushing; and thermodysregulation (the subjective sensation of being hot or cold without objective temperature change). Unsatisfiable itch, extensive exfoliation (referred to as “snow” by the patients due to the excessive skin shedding giving the appearance of snow falling on clothing or bedding), erythema with sharp cut offs at the palms (“red sleeves”), and “elephant skin” were also distinguishing. After establishing enrollment groups, we compared skin and blood markers by transcriptomics, shotgun metagenomics, and metabolomics (2). We later used cell and mouse modeling to argue that TSW possessed unique overexpression of mitochondrial complex I; this led to an overproduction of NAD+ and a breakdown of tryptophan into neurotoxic metabolites (2). The report led to reconsideration of the diagnosis by some (73, 74) and garnered lay press attention from reputable outlets (75, 76).

**Figure 2:**
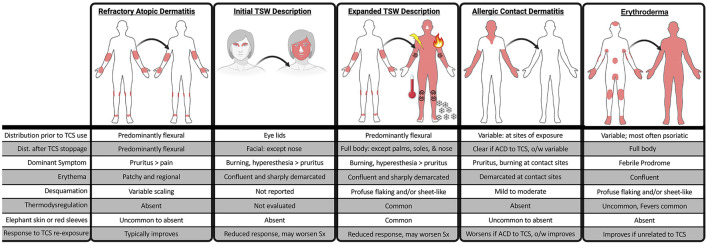
Overview of clinical features which distinguish topical steroid withdrawal (TSW) from untreated atopic dermatitis, allergic contact dermatitis (ACD), and Erythroderma. Expected clinical features are contrasted between refractory AD, the initial description of TSW [Rapaport and Rapaport (10)], the more recent description of TSW [Hsu et al. (1)], and ACD. For TSW, the icons are intended to highlight neuropathic pain (lightning bolt), burning (flame), thermodysregulation (thermometer), and profuse shedding (“snow”). TCS, topical corticosteroids; Sx, symptoms; Dist, distribution; o/w, otherwise. Image generated using a combination of BioRender, Photopea, and PowerPoint with royalty-free clipart.

The first Delphi distinguishing characteristics were published by a group led by Dr. Lio (1). The three-round process systematically identified some of the TSW-unique features based on the extensive clinical experience of 11 health care providers and 1 patient advocate: extensive exfoliation, hyperesthesia, “red sleeves,” “elephant skin,” and erythema extending beyond the patients underlying dermopathy. The report also details features which are shared with and differentiate TSW from other dermopathies. For example, based purely on cutaneous appearance, the disorder which may most closely mimic TSW is erythrodermic atopic dermatitis (also called generalized exfoliative dermatitis) (77). However, patients with erythrodermic AD lack the neurogenic and hyperesthesia symptoms of TSW, are acutely toxic with febrile hepatosplenomegaly, and have notable involvement of the palms and soles (78). Furthermore, erythrodermic AD is more often associated with psoriasis than atopy and is more common after the initiation of new medications compared to the discontinuation of TCS (78, 79) (Figure 2).

A second Delphi process identified consensus on treatment options (80). Other groups also presented distinguishing characteristics and/or called for more detailed research into TSW (81–86). Additionally, official recognition of TSW came from the NEA (87), the US National Institutes of Health (88), The Eczema Society of Canada (89), the British Association of Dermatologists, and the UK National Eczema Society (90). These acknowledgments followed updated labeling mandates from the UK government warning about the risk of TSW (3).

However, these developments have not precluded the same arguments against treating TSW as a distinct condition. Detractions continue to come in three overlapping categories: (a) insistence that some features of TSW overlap with other dermopathies and precludes the diagnosis of TSW; (b) claiming that any symptoms that might distinguish TSW are patient reported are thus inherently flawed; and (c) circular reasoning that the types of studies which may help distinguish TSW cannot be conducted until TSW is distinguished. The remainder of this review will address each of these in turn, focusing on specific examples of criticisms against the most recent mechanistic (2) and Delphi reports (1).

## Main fallacies of TSW denial

### Misdiagnosing a syndrome as a skin disease

As before, the most cited reasoning to consider TSW controversial rather than understudied is the established overlap with other dermopathies. This is particularly true for AD, which naturally waxes and wanes in ways that make it difficult to assign attribution of worsening underlying disease to medications vs. natural history. However, critics ignore the syndromic nature of TSW presentations. One example responding to the mechanistic findings in TSW raises concerns about potential “collider bias” by Tan et al. (91). The authors claim that if participants are selected for skin symptoms which collide with both TSW and exposure to topical corticosteroids (TCS), then any causal inference about TCS contributing to the symptoms is invalid. In essence, the claim is that since pruritic, erythematous skin might prompt TCS prescription, findings of pruritic and erythema in TSW cannot be considered distinct. These claims are the same as many prior, which appear to ignore the expansion of prior symptoms and additional symptoms. More broadly, a study into adverse medication effects will necessarily enroll individuals who have both taken the medication and had the symptoms for which the medication was prescribed; if an overlap between medical complications and underlying disease was considered inherently invalid, then analgesic headache and rhinitis medicamentosa would have been overlooked.

Tan et al. (91) offer an agreeable example of the correlation between use of angiotensin-converting enzyme (ACE) inhibitors and COVID-19 mortality; noting that age and comorbidities were underlying factors which drove both ACE use and COVID-19 mortality. However, this analogy fails when applied to matched control groups. Our TSW cohort was contrasted against patients of comparable demographics who had atopic dermatitis (AD) but did not report regression-derived TSW symptoms (2). Both groups therefore possessed the same proposed “colliders” of skin disease and TCS exposure, allowing comparisons between them. Furthermore, our work involved a separate cohort of healthy controls who received standardized TCS application (2). In this cohort, there was no confounding from pre-existing skin disease or prior high-potency steroid exposure. The replication of the same mitochondrial abnormalities under these controlled conditions strengthens the proposed TSW mechanism, even if the work cannot inform why most patients with prolonged TCS exposure do not develop TSW.

The conflation of TSW with undertreated AD implies that following scenarios are confusable (Figure 2):

Group A (refractory AD): patients with years of stable but fluctuating antecubital itch and eczematous rash who, upon discontinuation of TCS, experience a return of these exact symptoms on the same body sites.

Group B (original TSW description): patients with short term use of moderate to high potency TCS on the eyelids who, upon discontinuation of TCS, experience a marked change in the nature and intensity of their prior eyelid dermatitis which extends across the entire face.

Group C (current TSW description): patients with years of stable but fluctuating antecubital itch and eczematous rash who, upon discontinuation of TCS, experience new onset, full body itch and eczematous rash in addition to flushing, burning, neuropathic pain, thermodysregulation, and more.

Indeed, some patients could misrepresent refractory AD as “addiction” under the inaccurate logic that symptoms returning after TCS discontinuation represent addiction rather than continued disease. However, it is unlikely that any well-trained clinician could confuse these scenarios. One report noted delayed onset contact dermatitis on the face may mimic the initial description of TSW and suggested extending patch test evaluation beyond 7 days (92); but the authors did not present their case as evidence that TSW was non-existent. Yet claims that TSW may mimic allergic contact dermatitis (ACD) are only valid within the time frame of topical exposure (Figure 2). Patients with TSW may suffer protracted symptoms long after TCS discontinuation; with over 25% continuing to experience TSW-distinguishing symptoms longer than 1 year (38). Even if the initial erythema could not distinguish TSW from ACD, a history of protracted symptoms lasting for months-to-years after discontinuation certainly would. Ultimately, if providers wish to enhance patient trust they would be better served by explaining the differences between refractory AD, ACD, and TSW (1), rather than allowing erythema to be the lone symptom discussed.

Ultimately, Tan et al.'s (91) stated commitment to “uncovering the truth” through “robust statistics” is undermined by their recommendation for a placebo-controlled trial deliberately inducing TSW. On the one hand, while they are correct that a controlled withdrawal study would be more statistically robust, such a trial would be profoundly unethical. Within the 12-week timeframe proposed, the only available data suggests as many as 12% of participants could experience widespread symptom exacerbation and develop new pathology (12). While various therapies have been proposed in TSW (2, 20, 22, 33, 93), none are curative. Furthermore, TCS re-initiation is not an assured means of controlling the additional symptoms associated with TSW (1). Ethical studies using either controlled human-challenges or controlled withdrawals must offer curative treatment or re-initiation of prior control immediately after disease induction. In contrast, the trial the authors propose would intentionally expose participants to new pathologies without any reliable means of halting its progression. The authors also proposed an alternative approach of a longitudinal study following individuals who self-discontinue TCS (91) which is more ethically acceptable, but does not address their own stated concern for collider bias. Additionally, informed consent would require warning participants that abrupt discontinuation may cause serious harm, which itself introduces potential behavioral bias. Ultimately, the authors' proposed research approaches unethically conflate refractory AD with TSW, the very thing that is presented as a concern for inducing “steroid phobia” by the most critical publications.

### Disbelieving patient reported symptoms

Even when the syndromic nature of TSW is noted, a recurrent critique of TSW is that the distinguishing features are often patient reported. Critics suggest that recruitment through an advocacy group such as ITSAN adds bias by attracting individuals already concerned about TCS safety (42, 91). Again, recruitment for the diagnosis of eczematous skin disease would not impact the other specific symptoms. While patients with uncontrolled disease would be more likely to join a clinical trial, such selection bias would apply to any clinical study of any disease. More aptly however, while dermatology may benefit from physical presentations and biopsy data, patient reported symptoms are central to many fields (such as psychiatry). Even within dermatology however, patient reported symptoms are key to the diagnosis of AD. While appearance and distribution might be shortcuts, the widely used Hanifin and Rajka criteria (94) are unlikely to be met without subjective reporting. Among the major criteria, pruritus is purely subjective while relapsing-remitting chronicity and family/personal history of atopy are often patient reported. Subjectivity is also seen in sensitivity by environmental or emotional factors, food intolerance, intolerant to wool and lipid solvents, itching when sweating, and early age of onset (94). Notably, IgE biomarkers were consistent with subjective reports of new onset sensitivities in patients with TSW (95). While no assay for thermodysregulation, neuralgias, or hyperesthesia yet exists, this highlights that patient observations should not be dismissed as biased or subject to the whims of internet commentary.

### Circular logic on consensus

The final criticism of TSW as distinct is the self-fulfilling argument that studies into TSW cannot be performed without established diagnostic criteria, including studies which aim to establish diagnostic criteria. Such arguments overlap with the erroneous conclusions of misdiagnosis by implying that TSW cannot be studied against other dermatopathies, and therefore a true TSW cohort cannot be established (42, 47–49, 91). In addition to continuing to act as if the skin is the only organ system and ignoring the published TSW-distinguishing features, such claims are also ignorant of historical precedence. The first breakthrough in lupus erythematosus, in 1878, enrolled patients who met descriptions dating back to antiquity to better characterize the disease (96, 97). While the disease criteria have been subsequently amended numerous times and mechanistic understanding did not follow for decades, this example demonstrates that genuine intent to understand a disorder requires accepting initial uncertainty.

## True gaps in knowledge

Although we have articulated that most of the detractions against TSW are invalid, there are genuine areas of uncertainty which should be addressed in the future.

### Risk factors

Risk factor identification will be critical as currently, there are no known pharmacogenomic, microbiome, or environmental exposure factors which are known to predict who develops TSW and who does not. The only known risk predictors include duration, frequency, and potency of TCS. The cases of TSW presented in the literature each occurred in patients using TCS daily, or near daily, for more than 4–6 consecutive months (19–37, 98). No cases have been reported in patients using TCS with less frequency or duration. While cases are predominantly in those given moderate to high potency TCS, at least one case resulted from low (over the counter) potency (26); thus, potency alone does not provide assurance of safety from TSW. While slow tapering off TCS is recommended, the impact of such approaches on TSW risk is unclear (1).

Package inserts can provide dosing limitations such as body site-potency matching, limiting total exposure per day, and use < 2 consecutive weeks for anything more than 10% of body surface area; however, each of these cautions are caveated with the statement “unless instructed by a physician” (99). However, the AAD guidelines meant to inform physician instructions, only advice against “prolonged, inappropriate use” (16). Ascribing risk to “prolonged, inappropriate use” (16) or stating that TCS are safe when used “appropriately,” without defining such terms, suggests the blame lies with the patient rather than the instructions. This is especially problematic when protracted, daily use was explicitly recommended in formal treatment guidelines as recently as 2013 (13).

### Biomarkers

Although the Delphi (1) and/or regression-derived (2) descriptions of TSW are adequate for initiating clinical trials, there are no current biomarkers of TSW. While some have suggested trans-epidermal water loss (TEWL) be incorporated into the diagnostic approach, TEWL is non-specific and confoundable (100). The reported mitochondrial NAD+ abnormalities linked to TSW were identified using imaging mass spectrometry on biopsy tissue (2), which is available only in research settings. The concerns for a disruption of the cutaneous glucocorticoid receptors (40, 41) should be revisited using isotype controls and/or state of the art methods. The proposed hypothesis centered on nitric oxide production from pericytes (101) should also be evaluated if the technical challenges of assessing these cells *in vivo* could be overcome. Studies could co-enroll TSW mimickers like erythrodermic AD, which also has no established biomarkers or pathogenesis (79). Identifying urinary and/or blood biomarkers obtainable by standard clinical laboratories should be prioritized in future studies. Overall, both mechanisms and biomarker insights for TSW are under active investigation and require both validation of current proposals and expansion of research.

### Incidence

The only attempted derivation of TSW incidence in the medical literature extrapolated from surveys indicating that 12% of patients with AD had disease which was refractory to TCS treatment (12). Given that this survey came before wide-spread availability of biologics, the 12% figure contains patients with AD severity beyond the expected TCS efficacy. However, even if the rate of TSW were 1%, it would not meet the definition of “rare” as “seldom occurring or found” (102). Given the wide-spread use of TCS, TSW could be termed “unusual” (not normal or typical, different or strange in a way that attracts attention) (103) but the resultant totality of patients impacted would only be “seldomly found” by those wishing to avoid them. While it is apparent that the rate of TSW is lower in children (1), the need for assessing pediatric incidence and risk mitigation is even more pressing. One approach might involve accessing a centralized health record for patients who received greater than 6 months of TCS prescription, then perform chart reviews for TSW symptoms.

Although far lower for topical formulations, the absolute risk difference for shingles (zoster) complications with the use of oral Janus Kinase (JAK) inhibitors is 1.7% (104). Despite the realization that informing patients of such knowledge would increase the odds of patient refusal, we do not find any use of the term “phobia” applied to patients who decide that their ongoing symptoms do not justify the stated risks. In contrast, calls have been made to firmly establishing the risk against which benefits can be compared (105).

### Medical documentation

Risk mitigation, biomarker identification, and calculating incidence and prevalence each are best performed with adequate documentation of disease. Typically, this is performed using an International Classification of Disease (ICD) code, run by the World Health Organization (WHO) and managed within the US by the Center for Disease Control and Prevention (CDC). These codes are more frequently used for billing but are central to epidemiologic tracking (106). In 2026, the CDC announced that TSW would be incorporated into the ICD system under the codes L30.6 and T49.0X5S. While this will not go into effect until 2027, the ability to document TSW in a way that distinguishes it from other dermopathies will be central to bridging the remaining knowledge gaps.

### Treatment

To date treatment approaches for TSW have included: targeting Th2 cytokines (dupilumab) (20); targeting mitochondrial complex I (berberine, metformin, methylene blue, and co-enzyme Q12) (2, 107); T-cell suppression (ciclosporin) (93); JAK inhibition (ruxolitinib) (33); and traditional Chinese medicine (22). Each of these studies are small and lacked placebo controls. Other treatment options, such as cold atmospheric plasma (108), have been noted by patients but lack any formal assessment of efficacy or safety in TSW. Identification of new therapies as well as validation of any currently proposed treatments should be initiated and prioritized.

## Discussion

After examining the history of the debate surrounding TSW, we propose a shift in referring to TSW as “controversial” to instead refer to it as “poorly understood.” Similar retirement should befall the terms “steroid phobia” and “corticophobia,” whose adoption by academics seems the original failure point leading to the divide with patients. Failure to do so will continue to send the signal that academics do not take patient concerns seriously: no one should be diagnosed, treatments should only be repurposed from AD, no prevention should be attempted, and no studies should be performed. Other drugs have side effects which may dissuade patient use; but use continues partly because such concerns were studied, and the results were transparently reported (109). Those concerned with patients avoiding a potentially beneficial treatment should adequately communicate the differences between refractory AD and the syndromic nature of TSW. By better informing patients about the risks inherent to daily (or near daily) TCS use lasting more than 4–6 consecutive months (1, 80), providers can better reassure patients whose TCS needs fall well below such thresholds.

Additional treatment recommendations center on being both compassionate and curious about the patient's symptoms and acknowledge why patients may be angry at the medical system. Many will have been prescribed TCS for prolonged periods without receiving adequate warnings about limiting use frequency from their prescriber. Invalidating the patient concerns will cause further disengagement from the medical system, especially given that the system is responsible for the patient's injury.

Providers must inquire about symptoms beyond objective skin exams, especially neurogenic symptoms which distinguish TSW from other dermopathies. In addition, providers should inquire about psychological distress, which is high in TSW patients as evidenced by high frequency of reported social withdrawal, anxiety, depression, and suicidal ideation (38, 60). Providing compassionate care, and avoiding emotional invalidation, are key to addressing the mental health burdens of TSW.

If seeing a patient with suspected TSW for the first time, providers should obtain details on the history of body site distribution over the course of the patient's disease. Detailed histories including timelines of symptoms should be obtained since not every distinguishing feature of TSW is present at the same time or in every patient. Even if the skin appears consistent with the diagnosis of AD, inquiry into any rapid expansion of disease should be performed.

Finally, we recommend that the academies for medical specialties create formal processes for hearing from the patient advocacy groups which fall within their purview. Such programs will not assure agreement but would facilitate routine opportunities to close gaps between academics and advocates. Historical examples from the earliest days of the Human Immunodeficiency Virus (HIV) pandemic demonstrate how formal discussions between researchers and patient advocates can benefit both groups even without complete agreement (110). Had early concerns for TSW been given a platform with those labeling their concerns as “phobia,” perhaps recognition of the syndromic aspects could have prompted targeted research.

While online information will always carry risks of inaccuracies, better codifying both the TSW knowledge and the knowledge gaps might have offered a more robust information pool for content creators to reference. Counterintuitively, constructing a system to discuss concerns surrounding TCA is more important in the current era of expanded, non-steroidal treatment options. As more medications hit the market, the chances that some may present toxicities which arise only after prolonged use increase. Having a formal mechanism for patients and independent providers to raise concerns could inform research and diagnosis and would better assure disagreements offered by academic leaders were evidence-based.
